# Comparative evaluation of pyrolysis oils derived from lignocellulosic biomass and municipal solid waste as potential bitumen modifiers

**DOI:** 10.1007/s11356-026-38286-7

**Published:** 2026-10-09

**Authors:** Bhaswati Bora, Nikhil Saboo, Praveen Kumar, Sonal K. Thengane

**Affiliations:** 1 Department of Civil, Environmental and Architectural Engineering, University of Padua, Padua, 35131 Italy; 2 Department of Civil Engineering, Indian Institute of Technology, Roorkee, Uttarakhand 247667 India; 3 Department of Hydro and Renewable Energy, Indian Institute of Technology, Roorkee, Uttarakhand 247667 India

**Keywords:** Thermochemical conversion, Bio-bitumen, MSW-bitumen, Chemical characterization, Thermal behavior, Material characterization

## Abstract

The transition toward sustainable road construction has led to increased research on replacing conventional asphalt binders with bio-based and waste-derived alternatives. Bio-bitumen derived from lignocellulosic biomass and binders modified using domestic and industrial waste have shown significant potential in reducing greenhouse gas (GHG) emissions by up to 80%. Life cycle assessments have reported net-negative emissions for certain biomass-based binders. However, these alternative binders may alter the chemical and molecular compositions of the base bitumen, thereby affecting its physical characteristics and overall performance. Pyrolysis of biomass and non-recyclable waste is emerging as a promising alternative due to its chemical similarity to fossil fuel–based oils. Despite its potential, the performance of pyrolyzed oils is highly dependent on feedstock type and process parameters. This study investigates the chemical, thermal, and rheological properties of binders produced from lignocellulosic sources (pine needles, sawdust, and rice straw) and a waste source (municipal solid waste). Five percent pyrolyzed oil from each source was added to the base bitumen (VG 40 in this study) for laboratory investigation. Results show that pyrolysis oils are chemically compatible with bitumen, but the variations in molecular composition and their quantitative presence affect the binder characteristics. Among all the samples, pine needle (PN) feedstock and PN bio-oil exhibited a balanced chemical profile with lower polarity, contributing to improved compatibility with petroleum binders. PN bio-bitumen demonstrated the closest similarity to the base bitumen in terms of molecular structure, thermal stability, rheological and aging response. The study concludes that the performance of bio-bitumen is strongly influenced by the source of pyrolysis oil, with PN bio-bitumen showing comparatively promising results for further development.

## Introduction

The replacement of asphalt binder with sustainable alternatives, such as waste-derived and bio-based resources, is a rapidly growing area of research aimed at developing environmentally friendly road construction materials. Bio-bitumen produced from lignocellulosic biomass has been extensively studied over the past decade, offering a sustainable alternative to fossil fuel-based binders for road construction (He et al. 2023; Fini et al. 2012)*.* Lignocellulosic bio-binders are derived from plant-based biomass composed primarily of cellulose, hemicellulose, and lignin: components found in agricultural residues, forestry waste, and other plant materials. In parallel, the incorporation of domestic and industrial waste materials, such as waste plastics, waste engine oil, and waste cooking oil, has also contributed to the development of alternative, sustainable binders*.* In both cases, typical dosages of bio-oil or waste-derived materials used in modified binders range from 5 to 20% by weight of the total binder, depending on the specific material properties and target performance characteristics (He et al. 2023; Ahmed and Hossain 2020). Recent studies have reported that lignocellulosic biomass- and waste-derived binders can reduce CO₂ and greenhouse gas emissions by up to 80% compared with conventional bitumen (Khandelwal 2019; Ganguly et al. 2022). Conventional bitumen typically exhibits GHG emissions of approximately 450–500 kg CO₂-eq/ton, whereas bio-based and waste-derived binder systems have been reported to substantially reduce these emissions (Ganguly et al. 2022; Movilla-Quesada et al. 2021). Additionally, life cycle assessments (LCA) of bio-bitumen have reported net negative emission values when specific biomass sources are utilized (Zhou et al. 2021; Tokede et al. 2020). These findings are context-specific and cannot be generalized to the bio-binders investigated in this study without conducting dedicated LCA analyses accounting for the specific feedstocks, pyrolysis conditions, and geographic contexts considered here.

Although alternative binders offer sustainable solutions, modifying or replacing conventional bitumen alters its chemical composition. Bio-based materials are typically softer, which results in lower stiffness of bio-bitumen compared with conventional bitumen (Mehta and Saboo 2025). Therefore, external modifications to bio-bitumen are often recommended to enhance binder stiffness. Chemical characterization of bio-bitumen has shown an increase in phenolic compounds, indicating lower oxidation potential and reduced susceptibility to aging (Park et al. 2022). Zhang et al (2024) observed changes in molecular composition, including increases in saturates and aromatics fractions, following the addition of 7% bio-oil to base bitumen. In other studies (Ahmed and Hossain 2020; Al-Sabaeei et al. 2020), rheological analyses indicated lower production temperatures, improved low-temperature performance, and compromised high-temperature properties of bio-bitumen. Consequently, chemical modification of bio-oil or the use of polymers is commonly recommended to enhance the overall performance of bio-binders. While bio-bitumen offers a reduced carbon footprint and potential self-healing properties, its compatibility and long-term performance require molecular-level modification and stabilization.

In the case of waste-modified binders, such as those incorporating waste plastic and rubber, increased stiffness values are typically observed due to the presence of complex polymeric compounds in the waste materials (Hall and White 2021; Varun Kumar et al. 2024). Similarly, the molecular composition of these binders may vary, as polymeric waste materials often contain higher proportions of asphaltenes and resins. Xu et al (2022) reported improved high-temperature performance but reduced low-temperature performance when using polymeric waste materials. Other bio-based waste-modified binders have demonstrated performance comparable to that of lignocellulosic bio-binders, as reported in previous studies (He et al. 2023; Ahmed and Hossain 2020).

Pyrolysis oil has emerged as a viable candidate for use both as a sustainable asphalt binder replacement and as a functional modifier in pavement applications. It is produced through a thermochemical conversion process at temperatures ranging from 300 to 600 °C, which transforms biobased or non-recyclable waste into sustainable oil resources (Anshu et al. 2024; Zhou et al. 2021). Over the past decade, pyrolysis oil has been widely investigated as a substitute for fossil‑based oils because its chemical structure closely resembles that of petroleum‑derived counterparts, making it a promising sustainable binder alternative (Lu and Gu 2022). However, previous studies have reported inconsistent results for pyrolysis oils from different origins, highlighting the need for a systematic comparison across multiple feedstocks (Fini et al. 2017; Zhang et al. 2024). Wu and Hou (2024) reported that the incorporation of bio-oil into lignin-modified asphalt improved ductility, adhesion, fatigue resistance, and low-temperature cracking performance while significantly influencing binder rheology and microstructure. Guo et al (2024) investigated bio-oil/lignin composite-modified asphalt and observed excellent storage stability and favorable rheological characteristics, concluding that the modification process was predominantly physical in nature. More recently, Liu et al (2025) demonstrated that phenol-rich bio-oils produced from biomass waste could effectively retard oxidative aging of bitumen, reducing carbonyl formation and molecular growth while improving fatigue and low-temperature cracking resistance. These findings confirm that biomass-derived pyrolysis oils can enhance the sustainability and functional performance of asphalt binders. However, their effectiveness remains highly dependent on feedstock composition, chemical functionality, and molecular characteristics. Consequently, a comprehensive comparison of pyrolysis oils derived from different lignocellulosic feedstocks under identical production conditions remains necessary to establish source-performance relationships and identify the most suitable bio-oil candidates for asphalt applications.

Although biomass- and waste-derived pyrolysis oils have been previously investigated for asphalt modification, most studies focus on a single feedstock source. The novelty of the present work lies in the systematic comparison of pyrolysis oils derived from three lignocellulosic biomass sources (pine needle, rice straw, and sawdust) and one municipal solid waste source, all produced under identical pyrolysis conditions and evaluated at the same dosage level. Furthermore, the study integrates feedstock characterization with comprehensive binder-level analyses including FTIR, GPC, TGA, storage stability, rheological, and aging assessments, thereby establishing feedstock–performance relationships and identifying the most suitable pyrolysis oil source for asphalt modification.

## Materials and methods

### Materials

Bitumen is a complex hydrocarbon blend of asphaltenes, saturates, resins, and maltenes. Its combined viscoelastic and thermoplastic behavior makes it an ideal binder for flexible pavement applications in road construction. In this study, a viscosity graded (VG) binder, VG 40, was used for the production of bio-bitumen. As per IS 73:2013 (2013), VG 40 bitumen is generally used for very heavy traffic (over 30 msa) and high pavement temperature (over 45 °C) conditions. Biomass sources used for bitumen modification are predominantly derived from plant-based lignin, algal biomass or various bio-waste. In this context, pine needle (PN), sawdust (SD), and rice straw (RS), representing abundant lignocellulosic biomass resources, were selected as feedstocks in this study. Municipal Solid Waste (MSW) refers to the heterogeneous mixture of discarded materials generated from residential, commercial, institutional, and sometimes industrial sources. It typically includes biodegradable organics, plastics, paper, textiles, glass, and inert materials. The biomass and the MSW can be valorized through thermochemical or biochemical processes to recover energy or produce value-added products such as bio-oil, char, or syngas. When thermally treated, the organic fraction of the materials yields bitumen-like oxygenated compounds such as phenols, ketones, and acids, making it a promising candidate for bio-binder development in sustainable road materials.

### Pyrolysis oil production

For the production of pyrolysis oil from biomass sources and MSW, the raw materials were first dried to reduce their moisture content to below 10%. The dried materials were then shredded and crushed to a uniform fine particle size (≤ 2 mm) to ensure efficient heat transfer during pyrolysis. Subsequently, the crushed feedstock was heated at 105 °C for 1 h to completely eliminate residual moisture. Preheating the reactor feedstock enhances thermal efficiency and improves the oil yield from the dewatered material. A fixed-bed pyrolysis reactor was employed to produce pyrolysis oil under controlled conditions conducive to thermochemical conversion. The feedstock was introduced into the reactor via a screw feeder integrated into the system. A reaction temperature of 500 °C with a holding time of 30 min was selected based on the optimized operating conditions of the fixed-bed reactor and for maximizing bio-oil yield from lignocellulosic biomass (Zhou et al. 2021). To ensure a consistent comparison, identical pyrolysis conditions were applied to all feedstocks, enabling the observed differences in bio-oil composition and binder performance to be attributed primarily to feedstock characteristics rather than processing conditions. An inert atmosphere was maintained during the reaction by introducing nitrogen gas into the reactor at a flow rate of 0.2 L/min. The resulting bio-oil was condensed, separated, and collected in a receiving flask.

### Bio-bitumen and MSW-bitumen production

The modified bitumen was prepared by replacing 5% of the base binder (by weight) with pyrolysis oil. This substitution rate was intentionally selected as a fixed comparative dosage for two primary reasons. First, a uniform dosage across all feedstocks was necessary to isolate the effect of feedstock chemistry on binder performance, independent of concentration effects. Second, 5% was selected as a conservative starting point consistent with the lower bound of dosage ranges reported in the bio-asphalt literature (Ahmed and Hossain 2020; Fini et al. 2017), where initial binder-level screening studies commonly employ low substitution rates to avoid excessive rheological deterioration before mixture-level optimization.

**Fig. 1:**
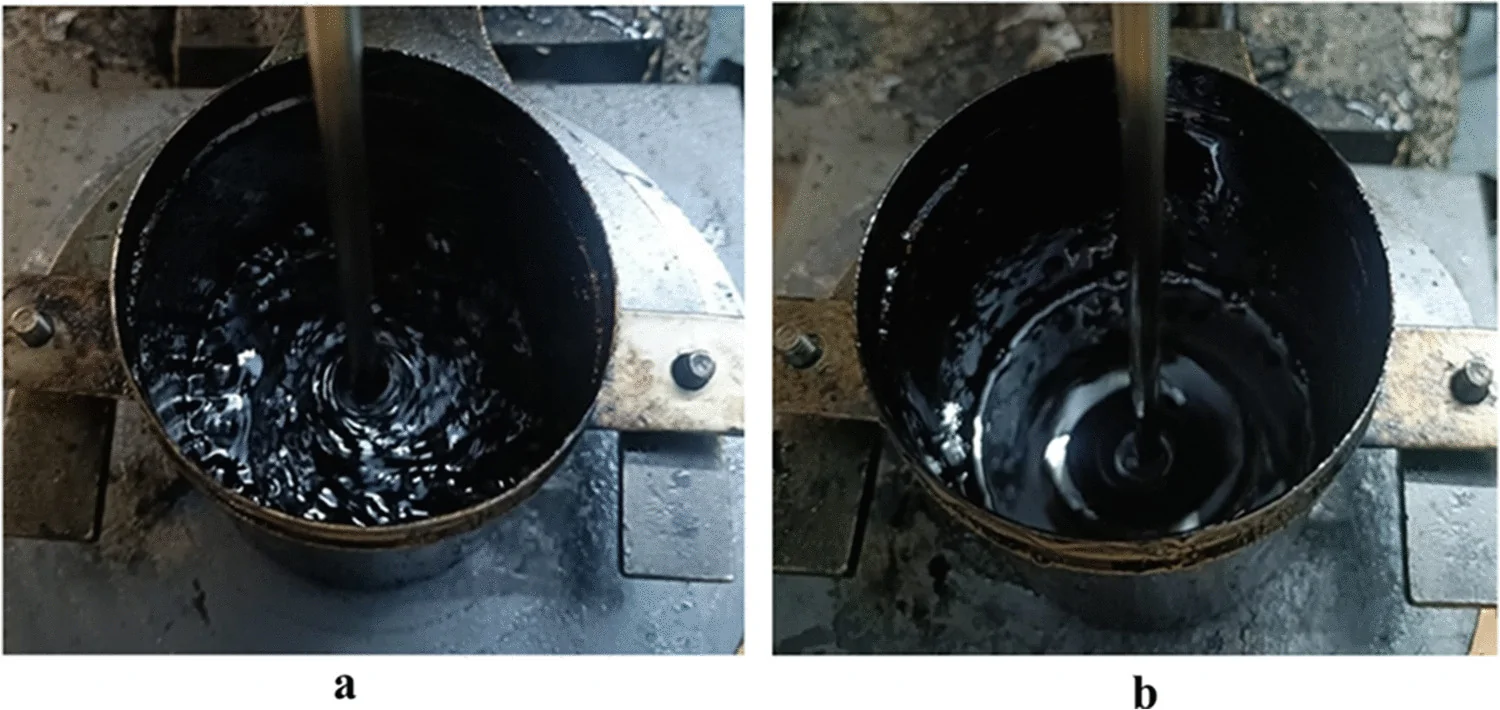
Production of bio-bitumen and MSW-bitumen

The bitumen modified with oils derived from PN, RS, and SD is collectively referred to as bio-bitumen, while the binder modified with oil from MSW is referred to as MSW-bitumen. The preparation procedures for both bio-bitumen and MSW-bitumen were developed through multiple trial blends in the laboratory. Bio-bitumen was produced by physically blending the pyrolysis oil with the base binder using a mechanical stirrer. Literature indicates that pyrolysis oils are generally compatible with conventional bitumen. Accordingly, the blending was conducted at 135 °C for 20 min at a stirring speed of 1000 rpm. These relatively mild blending conditions were chosen to preserve the structural integrity of the organic fractions in the pyrolysis oils. Due to the moisture content in the pyrolysis oils, foaming was observed during the heating and blending process (see Fig. 1a). However, the foaming subsided toward the end of the blending process, resulting in a uniform mixture (see Fig. 1b).

### Proximate and ultimate analyses

Proximate and ultimate analyses are used to characterize the composition of fuels like coal, biomass, or bio-oil. In proximate analysis, the focus is on basic components such as moisture, volatile matter, ash, and fixed carbon. This is usually done by heating the sample in controlled steps and measuring weight changes to see what burns off and what remains. Ultimate analysis, on the other hand, digs deeper into the chemical structure of the fuel. It measures elements such as carbon, hydrogen, nitrogen, sulfur, and oxygen, often using specialized instruments like CHNS analyzers. While proximate analysis gives a quick snapshot of how the fuel might behave during combustion, ultimate analysis provides detailed information needed for energy calculations and emission predictions. The compositions of PN, RS, SD and MSW feedstocks are investigated through proximate and ultimate analyses in this study.

### FTIR studies

Fourier Transform Infrared (FTIR) Spectroscopy is commonly used to characterize the functional groups and chemical structure of pyrolysis oil. FTIR has proven to be a powerful technique for identifying organic compounds and functional groups present in asphalt binders (Bora and Das 2020; Lamontagne et al. 2001). It provides a molecular fingerprint of various organic constituents, such as phenols, ketones, carboxylic acids, and aldehydes, which are typical products of biomass pyrolysis. By analyzing absorption peaks in the infrared spectrum, FTIR enables the assessment of the reactivity, aging behavior, and compatibility of pyrolysis oil with bitumen, making it a valuable tool for evaluating the potential of different oils as suitable modifiers (Yao et al. 2015; Dong et al. 2018). In this study, the organic compounds and structural configurations were identified from the IR spectra generated by the vibrational modes of molecular bonds at varying infrared wavelengths. The spectra were recorded in the range of 4000–400 cm⁻^1^, with the samples scanned at a resolution of 4 cm⁻^1^. The aging index, aromaticity, and moisture sensitivity of the binders were subsequently estimated based on the FTIR spectral data.

1 $$\begin{array}{c}{I}_{\mathrm{C}0}= \frac{{I}_{1700}}{{I}_{\mathrm{R}\mathrm{E}\mathrm{F}}}\end{array}$$

2 $$\begin{array}{c}{I}_{\mathrm{S}0}= \frac{{I}_{1600}}{{I}_{\mathrm{R}\mathrm{E}\mathrm{F}}}\end{array}$$

3 $$\begin{array}{c}{I}_{\mathrm{O}\mathrm{H}}= \frac{{I}_{3440}+ {I}_{1340}}{{I}_{\mathrm{R}\mathrm{E}\mathrm{F}}}\end{array}$$

4 $$\begin{array}{c}{I}_{\mathrm{A}\mathrm{R}\mathrm{O}\mathrm{M}\mathrm{A}\mathrm{T}\mathrm{I}\mathrm{C}\mathrm{I}\mathrm{T}\mathrm{Y}}= \frac{{I}_{1635}}{{I}_{\mathrm{R}\mathrm{E}\mathrm{F}}}\end{array}$$

The modification and oxidation quantification in binders can be investigated using the indices presented in Eqs. (1–4). The carbonyl index (${I}_{\mathrm{C}0})$ indicates oxidation due to ketones, aldehydes, and carboxylic acids formation. The sulfoxide index (${I}_{\mathrm{S}0})$ tracks the oxidation of sulfur-containing compounds. The aromatic index (${I}_{\mathrm{A}\mathrm{R}\mathrm{O}\mathrm{M}\mathrm{A}\mathrm{T}\mathrm{I}\mathrm{C}\mathrm{I}\mathrm{T}\mathrm{Y}})$ reflects the presence of stable aromatic rings. The hydroxyl index (${I}_{\mathrm{O}\mathrm{H}})$ measures the presence of alcohol and phenol groups. The indices are calculated by normalizing the area of the respective functional-group peaks to the reference peak at approximately 1460 cm⁻^1^, corresponding to the aliphatic C–H bending vibration. This peak was selected as the reference because it remains relatively stable during binder modification and aging and is therefore widely used as an internal reference for FTIR-based asphalt characterization (Bora and Das 2020; Lamontagne et al. 2001).

The chemical quantification was conducted using the transmission mode to scan both the pyrolysis oils and binder samples. The binder samples were prepared in solution form using carbon disulfide (CS₂) as the solvent at a concentration of 16.67 wt/vol%. Approximately 10 µL of the binder solution was drop-cast onto potassium bromide (KBr) pellets using a micropipette, yielding a sample volume in the range of 1–10 µL. For the pyrolysis oils, FTIR spectra were directly obtained by drop-casting the oils onto KBr pellets. Both the binder solutions and pyrolysis oil samples were dried in a desiccator prior to scanning to eliminate moisture interference.

### GPC studies

Gel Permeation Chromatography (GPC) is a fast and effective method for fractionating asphalt binders based on their hydrodynamic volume in solution, which correlates with molecular weight. A chromatogram of detector response versus elution time or volume is generated for the solvent-based solution. The detector response is then converted into molecular weight concentration using appropriate calibration methods. Molecular weight fractions are typically categorized into large molecular weight (LMW), mid-range molecular weight (MMW), and small molecular weight (SMW) fractions. LMW components primarily include asphaltenes, resins, and lignin; MMW represents aromatic and heavy oil fractions; and SMW corresponds to saturates and other lightweight fractions (Li et al. 2022). Common molecular weight distribution parameters such as number-average molecular weight (*M*_*n*_) and weight-average molecular weight (*M*_w_) obtained from GPC analysis show strong correlations with the molecular fractions present in bitumen (Zhang et al. 2019). *M*_*n*_ represents the average molecular weight sensitive to all low-molecular-weight components molecules and *M*_w_ is sensitive to the weight of heavier molecules. A lower polydispersity index (*M*_w_/*M*_*n*_) suggests a homogeneous molecular weight distribution and molecular sizes. These distribution parameters are selected based on their sensitivity to variations in the molecular weights of bitumen components. Bitumen typically exhibits multimodal peaks in GPC output, reflecting its composition of multiple molecular weight fractions (Negulescu and Balamurugan 2013). The molecular weight parameters and corresponding molecular fractions estimated in this study are presented as follows:

5 $$\begin{array}{c}{M}_{n}= \frac{\sum \left({N}_{i}.{M}_{i}\right)}{\sum {N}_{i}}\end{array}$$

6 $$\begin{array}{c}{M}_{w}= \frac{\sum \left({N}_{i}.{M}_{i}^{2}\right)}{\sum \left({N}_{i}.{M}_{i}\right)}\end{array}$$

7 $$\begin{array}{c}\mathrm{P}\mathrm{o}\mathrm{l}\mathrm{y}\mathrm{d}\mathrm{i}\mathrm{s}\mathrm{p}\mathrm{e}\mathrm{r}\mathrm{s}\mathrm{i}\mathrm{t}\mathrm{y} \mathrm{I}\mathrm{n}\mathrm{d}\mathrm{e}\mathrm{x}= \frac{{M}_{w}}{{M}_{n}}\end{array}$$

8 $$\begin{array}{c}\mathrm{L}\mathrm{M}\mathrm{W}= \frac{{\mathrm{A}\mathrm{r}\mathrm{e}\mathrm{a}}_{\mathrm{L}\mathrm{M}\mathrm{W}}}{{\mathrm{A}\mathrm{r}\mathrm{e}\mathrm{a}}_{\mathrm{T}\mathrm{o}\mathrm{t}\mathrm{a}\mathrm{l}}}\times 100\end{array}$$

9 $$\begin{array}{c}\mathrm{M}\mathrm{M}\mathrm{W}= \frac{{\mathrm{A}\mathrm{r}\mathrm{e}\mathrm{a}}_{\mathrm{M}\mathrm{M}\mathrm{W}}}{{\mathrm{A}\mathrm{r}\mathrm{e}\mathrm{a}}_{\mathrm{T}\mathrm{o}\mathrm{t}\mathrm{a}\mathrm{l}}}\times 100\end{array}$$

10 $$\begin{array}{c}\mathrm{S}\mathrm{M}\mathrm{W}= \frac{{\mathrm{A}\mathrm{r}\mathrm{e}\mathrm{a}}_{\mathrm{S}\mathrm{M}\mathrm{W}}}{{\mathrm{A}\mathrm{r}\mathrm{e}\mathrm{a}}_{\mathrm{T}\mathrm{o}\mathrm{t}\mathrm{a}\mathrm{l}}}\times 100\end{array}$$

Prior to sample analysis, the GPC system was calibrated using monodisperse polymer standards, and a calibration curve relating elution volume to logarithmic molecular weight was established under tetrahydrofuran (THF) elution conditions. Molecular weight distribution parameters were subsequently determined from the calibrated chromatograms obtained using a refractive index (RI) detector. The *M*_*n*_, *M*_w_, and *M*_w_/*M*_*n*_ were calculated from the chromatographic data using the standards-based calibration method. The samples were prepared by dissolving 10 mg of binder in 10 mL of THF, maintaining a consistent concentration of 1 mg/mL across all sample solutions. A 100 μL portion of each solution was injected into the GPC column, with an analysis duration of 40 min and a flow rate of 1.000 mL/min.

### Thermal stability using TGA

The volatilisation of lightweight compounds, moisture removal, and decomposition kinetics of bitumen can be identified and analyzed using a thermogravimetric analyzer (TGA). Therefore, the thermal decomposition behavior, moisture release pathways, asphaltene degradation temperature, and residue characteristics were investigated using TGA. The decomposition kinetics of the samples were determined from the TGA curve, and the derivative thermogravimetric (DTG) curve was analyzed to estimate the maximum mass loss and identify the peak decomposition temperatures of the compounds (Mehta and Saboo 2025; Varun Kumar et al. 2024). Peak analysis of the DTG curve was also conducted to quantify mass loss and estimate the decomposition temperatures of the compounds based on their molecular weights (Yu et al. 2016). For TGA analysis, approximately 10 mg of binder sample was tested from 30 to 800 °C under inert conditions, with a heating rate of 10 °C/min.

### Storage stability

Phase separation is a potential concern in both bio-bitumen and MSW-bitumen systems. Therefore, segregation analysis of the prepared samples should be conducted to assess their storage stability and homogeneity. Due to the volatilisation and oxidation reactions of bio-bitumen at elevated temperatures, the storage stability of the samples was evaluated at 140 °C, as suggested by Zhang et al (2018). The samples were conditioned at 140 °C for 48 ± 4 h in aluminum tubes measuring 136.7 mm in length and 25.4 mm in diameter. After conditioning, the samples were cooled to − 4 °C for 4 h, and the aluminum tubes were then cut into three equal parts. The top and bottom sections were tested to assess phase separation (Deb et al. 2025).

Phase segregation due to thermal effects was evaluated using the softening point (SP). The difference in softening points between the top and bottom sections was determined using the ring-and-ball method, in accordance with ASTM D36 (2021) (see Eq. 11). Rheological tests were also conducted to investigate phase segregation by measuring the complex shear modulus (G*) and phase angle (*δ*) of the top and bottom sections (see Eq. 12). G* and *δ* in Eq. 12 represent the complex shear modulus and phase angle of the binder when measured at specific conditions using a dynamic shear rheometer (DSR).

11 $$\begin{array}{c}\Delta \mathrm{S}\mathrm{P}=\left|{\mathrm{S}\mathrm{P}}_{\mathrm{u}\mathrm{p}\mathrm{p}\mathrm{e}\mathrm{r}}-{\mathrm{S}\mathrm{P}}_{\mathrm{l}\mathrm{o}\mathrm{w}\mathrm{e}\mathrm{r}}\right|\end{array}$$

12 $$\begin{array}{c}\mathrm{S}\mathrm{e}\mathrm{g}\mathrm{r}\mathrm{e}\mathrm{g}\mathrm{a}\mathrm{t}\mathrm{i}\mathrm{o}\mathrm{n} \mathrm{r}\mathrm{a}\mathrm{t}\mathrm{i}\mathrm{o}= \frac{{{G}^{*}/\mathrm{s}\mathrm{i}\mathrm{n}\delta }_{\mathrm{u}\mathrm{p}\mathrm{p}\mathrm{e}\mathrm{r}}}{{{G}^{*}/\mathrm{s}\mathrm{i}\mathrm{n}\delta }_{\mathrm{l}\mathrm{o}\mathrm{w}\mathrm{e}\mathrm{r}}}\end{array}$$

### Rheological study

The G* and *δ* of the samples were measured using a DSR for rheological analysis. A frequency sweep test was conducted on all binder samples. The test was performed over a temperature range of 10 to 80 °C at 10 °C intervals. An 8 mm spindle with a 2 mm gap was used to obtain G* and *δ* values from 10 to 30 °C, while a 25 mm spindle with a 1 mm gap was used for measurements from 40 to 80 °C. The frequency sweep was conducted within the linear viscoelastic (LVE) region using a fixed strain of 0.1%, with frequencies ranging from 100 to 0.1 rad/s. Data collected at different temperatures were compiled to generate master curves using the time–temperature superposition principle (TTSP). Reference temperatures of 20 °C, 40 °C, and 70 °C were selected for constructing the master curves of the binders under study. The shift factors at various temperatures were calculated using the Arrhenius equation (see Eq. 13), and the master curves for complex shear modulus and phase angle were modelled using the Christensen–Anderson and Marasteanu (CAM) model (see Eqs. 14 and 15) (Yusoff et al. 2011; Liu et al. 2022). The CAM model facilitates the development of continuous and smooth master curves for both G* and *δ*, enabling robust extrapolation and interpolation of rheological data beyond the limits of experimental measurements. This approach provides valuable insights into the stiffness and viscous characteristics of bitumen and is applicable to a wide spectrum of binders, including bio-based binders.

13 $$\begin{array}{c}\mathrm{log}{\alpha}_{T}=C\left(\frac{1}{T}-\frac{1}{{T}_{r}}\right)\end{array}$$

where *α*_*T*_ is the shift factor, *T* is the test temperature in Kelvin (K), and *T*_*r*_ is the reference temperature in Kelvin (K).

14 $$\begin{array}{c}\left|{G}^{*}\right|= {G}_{g}{\left(1+{\left(\frac{{f}_{c}}{f}\right)}^{\vartheta }\right)}^{-\frac{\omega }{\vartheta }}\end{array}$$

where G* is the complex shear modulus, *f*_*c*_ is the reduced frequency, and *ω* and *v* are the shape parameters.

15 $$\begin{array}{c}\delta = \frac{90\omega }{\left(1+{\left(\frac{{f}_{c}}{f}\right)}^{\vartheta }\right)}\end{array}$$

where *δ* is the phase angle.

### Binder aging

Short‑term aging of the binders was conducted using the Rolling Thin Film Oven (RTFO) procedure in accordance with ASTM D2872. In this method, the binder is poured into standard glass RTFO bottles and rotated in a horizontal oven while being exposed to a temperature of 163 °C for 85 min under a continuous stream of heated air. The combination of elevated temperature, thin‑film exposure, and forced air flow is designed to simulate the volatilization of lighter hydrocarbons and the early‑stage oxidative reactions that occur during hot mix asphalt production, storage, and field compaction. Following RTFO treatment, long‑term aging was conducted using the Pressure Aging Vessel (PAV) in accordance with ASTM D6521. In the PAV process, the RTFO‑aged binder is placed in shallow stainless‑steel pans to create a uniform thin film and subjected to an elevated pressure of 2.1 MPa (21 bar) and a temperature of 100 °C for a duration of 20 h. These conditions accelerate oxidative reactions by increasing the oxygen partial pressure, thereby simulating the gradual hardening that typically occurs in 5–10 years of field service.

## Results

### Compositional characteristics of biomass from different feedstocks

The proximate and ultimate analyses of PN, RS, SD, and MSW feedstock reveal clear variations that directly influence the suitability of each material for bitumen modification (refer to Table 1). Among the four feedstocks, PN shows the most favorable overall composition, with the lowest ash content (3.11%), relatively low moisture (4.68%), and the highest percentage of carbon (45.01%) and hydrogen (5.78%). These characteristics suggest a lower inorganic load and reduced polarity, both beneficial for binder compatibility and aging resistance. MSW exhibits moderately high carbon (42.50%) and hydrogen (5.60%) contents, comparable to PN, but shows elevated ash (10.65%) and the presence of sulfur (0.15%), the latter being absent in the biomass-derived oils. These factors point to a need for further purification (e.g., de-ashing) before binder application. RS and SD show considerably higher oxygen contents (44.33–45.22%) and the highest ash levels (10.85–12.69%), indicating a greater presence of oxygenated functional groups and inorganics. These properties are typically associated with increased acidity, moisture sensitivity, and accelerated oxidative aging in bitumen systems.

**Table 1:** Proximate and ultimate analysis for compositional analysis of the biomass feedstock

Property	PN	RS	SD	MSW
*Proximate analysis (average) (PA)*				
Average moisture content (%)	4.68	4.20	5.16	6.28
Average volatile mass (%)	73.80	64.30	64.03	68.86
Average fixed carbon (%)	18.40	18.80	19.96	14.21
Average ash content (%)	3.11	12.69	10.85	10.65
*Ultimate analysis (UA)*				
Carbon	45.01	33.37	37.52	42.50
Hydrogen	5.78	4.69	4.13	5.60
Nitrogen	0.77	0.72	0.79	0.60
Sulfur	0	0	0	0.15
Oxygen	40.65	44.33	45.22	40.50

Footnote* received basis for PA and dry basis for UA

#### Derived Van Krevelen indicators (H/C and O/C ratios)

Molar H/C and O/C ratios were calculated using elemental mass fractions from Table 1. These indicators are commonly used for classifying the relative saturation (H/C) and oxygenation/polarity (O/C) of carbonaceous materials. PN exhibits the most favorable profile with H/C = 1.541 and O/C = 0.677, indicating a relatively hydrogen-rich and less oxygenated material. MSW follows closely (H/C = 1.581; O/C = 0.715), although its higher ash and sulfur require consideration. RS and SD show substantially elevated oxygenation (O/C ≈ 0.90–1.00), indicating more polar species, consistent with their higher measured oxygen contents. The lower O/C ratios of PN and MSW strongly suggest reduced polarity, lower acidity, and better compatibility with hydrophobic petroleum binders, which is an observation consistent with patterns widely reported in pyrolysis literature.

#### Estimated higher heating value (HHV)

To further assess the energetic quality and potential refining behavior of the feedstocks, the higher heating value (HHV) was estimated using the Channiwala–Parikh elemental correlation linking the HHV to C/H/O/N/S/ash composition. PN shows the highest estimated HHV (18.24 MJ/kg), reflecting its higher C and H contents and low ash. MSW follows with 17.03 MJ/kg, again benefiting from relatively high C and H values. SD and RS, with higher oxygenation, exhibit significantly lower HHV values (13.05 and 12.31 MJ/kg, respectively). These differences align with the well‑established relationship between oxygen content and energy density in biomass, that is, higher oxygenation lowers the HHV by increasing the proportion of non‑combustible functional groups.

Based on the combined assessment of O/C ratio, H/C ratio, ash content, HHV compositional indicators, PN emerges as the most promising candidate for asphalt binder modification due to its low polarity, low ash content, and highest energy density. MSW presents a viable secondary option, though pretreatment to reduce ash and sulfur may be necessary. RS and SD are likely to require additional upgrading (e.g., de-ashing, esterification, mild hydro-deoxygenation) to compensate for their high oxygen content and substantial inorganic material, which may otherwise compromise moisture resistance, oxidative stability, and compatibility with petroleum binders.

### FTIR analysis

#### Pyrolysis oil analysis

**Fig. 2:**
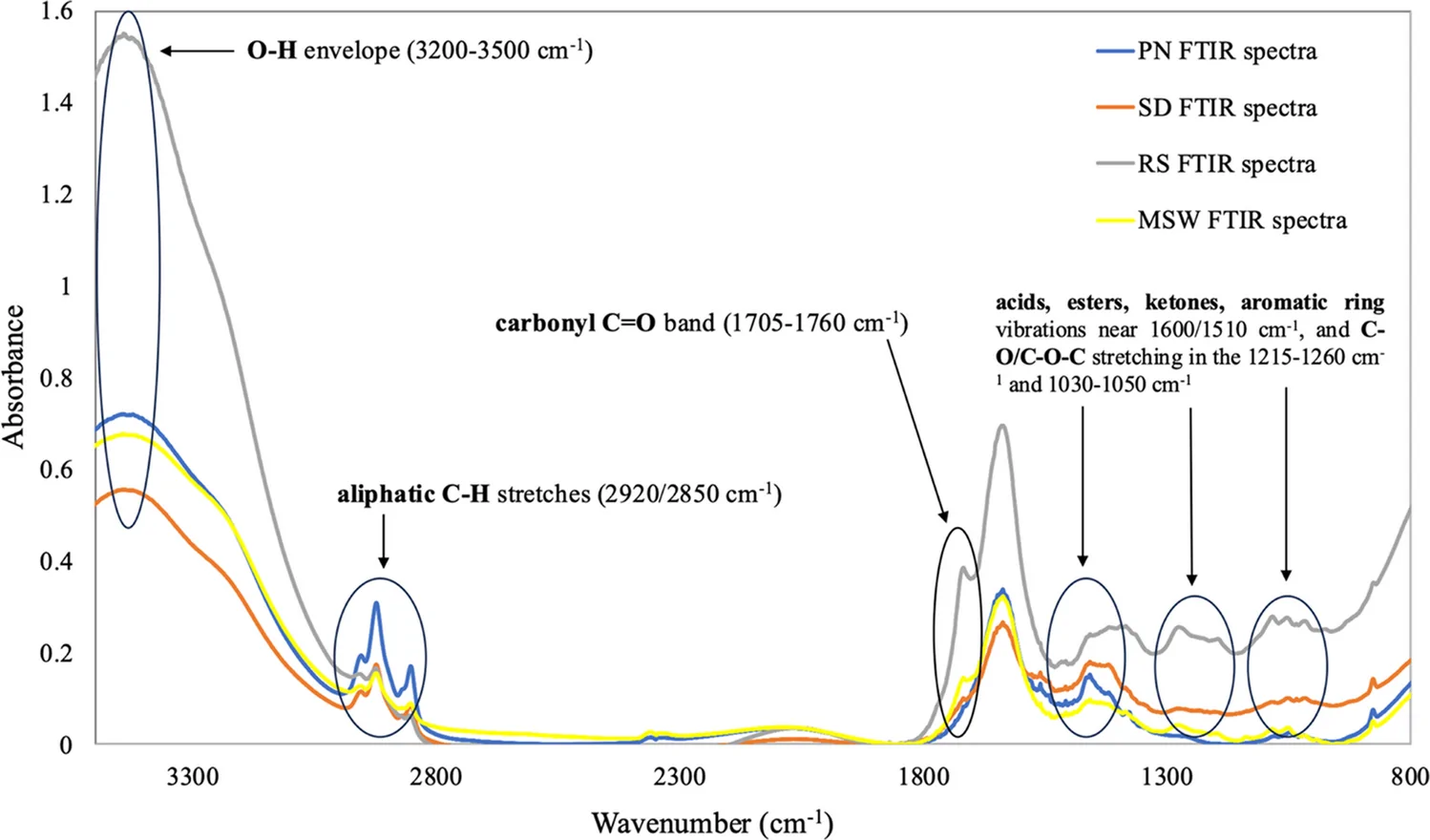
FTIR absorbance peaks of the pyrolysis oils

The FTIR spectra of the four pyrolysis oils exhibit characteristic functional‑group regions commonly observed in lignocellulosic and mixed‑waste pyrolysis liquids (Fig. 2). The broad O − H stretching band (3200–3550 cm⁻^1^) is most intense in the RS sample, followed by PN and MSW, with SD presenting the weakest response. This strong O − H envelope in RS indicates an elevated concentration of hydrogen‑bonded phenolic and alcoholic fractions, a typical signature of carbohydrate‑derived oxygenates, and aligns with its higher polarity and acidity. The aliphatic C–H stretching bands near 2920 and 2850 cm⁻^1^ are more prominent in PN and MSW than in RS or SD, suggesting a greater abundance of aliphatic chain structures in these two oils. Such aliphatic enrichment, often associated with lignin‑derived aromatics coexisting with aliphatics in PN and with plastic‑derived hydrocarbons in MSW, can enhance compatibility with the hydrophobic bitumen phase in binder applications. The C = O carbonyl band (1760–1705 cm⁻^1^) appears strongest in RS, consistent with high levels of carboxylic acids, esters, and ketones, whereas PN and MSW show moderate intensities and SD slightly lower. Aromatic skeletal vibrations near 1600 cm⁻^1^ and 1510 cm⁻^1^ are more distinct in PN, and to a lesser extent in SD, reflecting their lignin‑rich origin and higher phenolic content. Although the increased aromaticity enhances binder–aggregate adhesion, it must be carefully balanced with the oxygenated functional groups present in the oils, which can promote oxidative susceptibility. Finally, the C–O/C–O–C stretching regions (1260–1215 and 1050–1030 cm⁻^1^) are noticeably more intense in RS, indicating higher concentrations of ethers, alcohols, and anhydrosugar‑derived compounds. These oxygenated groups are known to increase polarity and reduce storage stability, reinforcing the need for pretreatment when such oils are incorporated into bituminous systems.

Overall, the FTIR features of each pyrolysis oil align closely with the compositional expectations derived from their feedstock origins. The PN oil shows a balanced chemical profile, with moderate O–H intensity, clear aliphatic C–H stretches, visible carbonyl peaks, and distinct aromatic bands. These traits correspond with its higher carbon and hydrogen content and low ash levels, suggesting lower polarity and better compatibility with asphalt binders, although this must be validated through rheological and aging tests. The RS oil, by contrast, exhibits dominant O–H, C = O, and C–O bands, confirming that it is highly oxygenated and strongly polar. Such chemistry often necessitates pretreatment such as de‑ashing, esterification, or mild hydrodeoxygenation to control acidity and moisture sensitivity before binder blending (Liu et al. 2025; Tabaković et al. 2023). SD displays an intermediate chemical character between PN and RS, with noticeable oxygenated functionalities but lower intensities than RS, along with some aromatic features. Moderate upgrading may therefore be required for SD‑derived oils to achieve binder‑level performance. MSW oil presents a distinct signature, characterized by stronger aliphatic C–H bands and moderate O − H and C = O responses, reflecting a more hydrocarbon‑like composition. However, owing to feedstock heterogeneity and the common presence of plastics and trace heteroatoms in MSW streams, additional care must be taken regarding de‑ashing, drying, and the potential presence of halogenated species. TG‑FTIR analyses of MSW in the literature frequently report HCl release from PVC, underscoring the need for proper pretreatment before incorporation into bituminous mixtures (Liu et al. 2025; Ephraim et al. 2023).

#### Analysis of base and modified binders

**Fig. 3:**
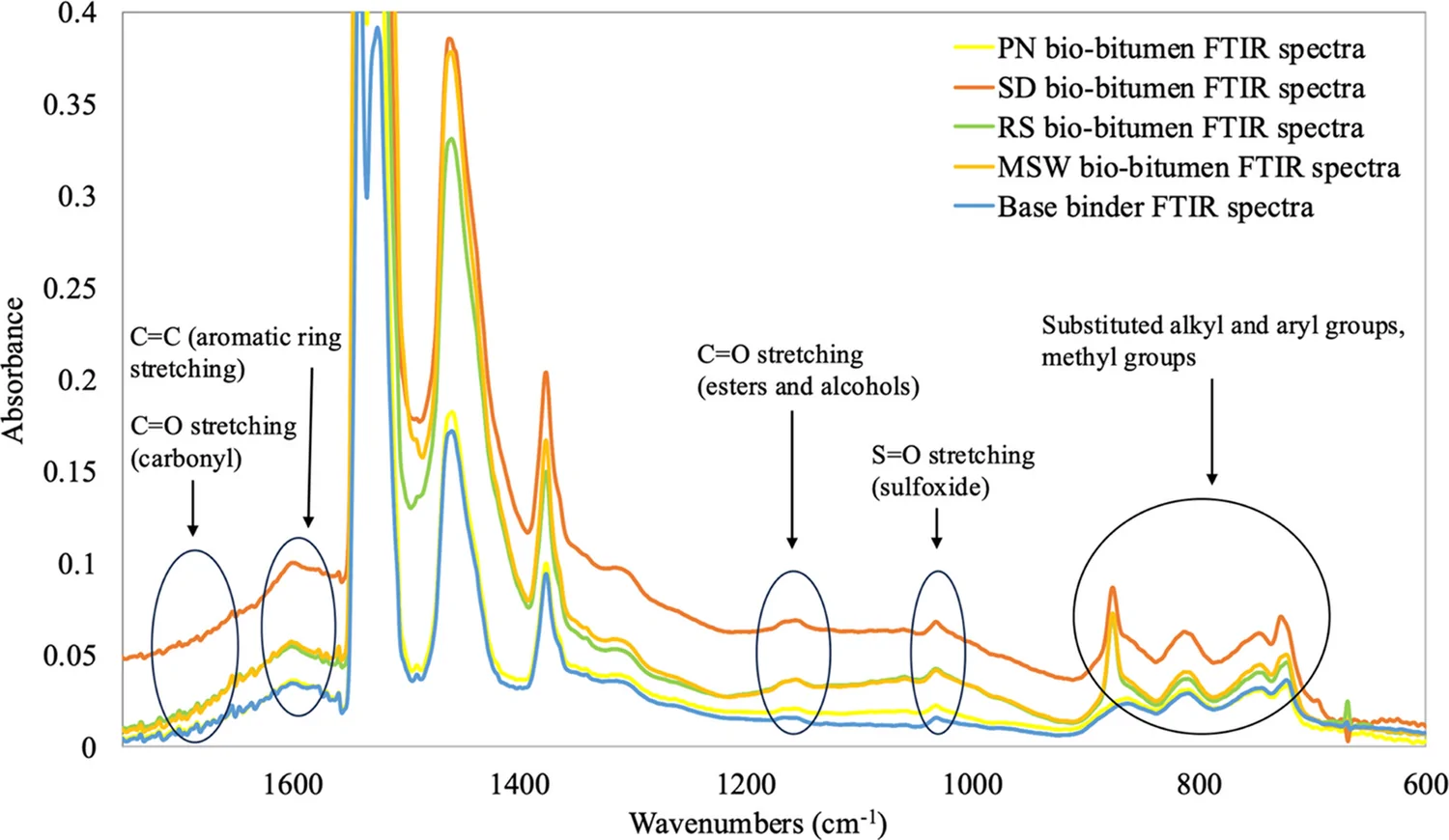
FTIR absorbance peaks of the binder samples

The bio-bitumen and the MSW-bitumen have higher and enhanced peaks than the base bitumen due to the presence of higher intensity peaks in the pyrolysis oil (refer to Fig. 3). The areas corresponding to the region 1200–1300 cm⁻^1^ showed enhanced peaks of alcohols and esters on C–O stretching and enhanced peaks of sulfoxides on S = O stretching. In the 1400–1600 cm⁻^1^, minor peak shifts are identified in the modified binders. This is due to hydrogen bonding or degradation of the polar compounds to stable compounds. The peaks in the region 1100–1300 cm⁻^1^ are broader, unlike the sharp peaks in the base bitumen, indicating hydrogen bonding and complex compounds with overlapping functionalities. Additionally, higher ketonic and cyclic compounds incorporating nitrogen and sulfur functionalities are identified in the 1610–1775 cm⁻^1^ range. The intensity of the peak identified in the region 700–900 cm⁻^1^ varies from the base bitumen due to the different substitution patterns or side chains (substituted with alkyl and aryl groups, including methyl groups) of the C–H out-of-plane bending from aromatic substitution. All binder samples exhibit both saturated alkanes and unsaturated alkenes. Characteristic peaks for straight-chain aliphatic hydrocarbons (alkanes) are identified at 2920 cm⁻^1^ (C–H asymmetric stretching) and 2850 cm⁻^1^ (C–H symmetric stretching).

The comparative interpretation of the spectra reveals differences between the base bitumen and the modified binders. The base bitumen is mostly hydrocarbon‑driven, with minimal contributions from oxygenated functional groups except those related to oxidative aging. The modified binders, however, show a chemical architecture rich in oxygen‑containing groups, especially esters and hydroxyls, resulting in substantially higher polarity. These differences are made evident by contrasting the strong ester carbonyl band at 1740 cm⁻^1^ in modified binders with its weak counterpart in the base bitumen. Similarly, modified binders exhibit more pronounced C–O absorptions and a noticeable O–H band (3200–3500 cm⁻^1^) not typically present in the base bitumen. This difference indicates that the carbonyl absorption for modified binders is primarily intrinsic (due to esters), whereas in asphalt it is extrinsic (associated with oxidation). Thus, carbonyl intensity cannot be directly compared between the two materials without considering its underlying chemical origin and oxidation.

The intensities of the identified peaks appear similar for the PN bio-bitumen and the base bitumen but lower than the other modified bitumen. Similarly, the O–H stretching region shows similar peak intensities for the PN bio-bitumen and base bitumen, whereas the RS bio-bitumen exhibits the highest alcohol peak intensity, suggesting higher hydroxyl content. An overall increase in the polar functionalities and oxidation compounds (O–H, C = O, S = O, and C–O) is mostly identified in the modified bitumen as compared to the base bitumen. Bitumen modifications cause peak shift, intensity change, and thermal degradation reactions during production. However, there is no major chemical modification on pyrolysis oil addition to the base bitumen. Therefore, the modification process is primarily physical in nature. It should be noted that compatibility analysis in this study is not based solely on FTIR analysis but are further supported by GPC molecular weight distribution and intrinsic viscosity data, storage stability testing results, and TGA thermal decomposition profiles, collectively providing a multi-technique basis for compatibility assessment.

The FTIR indices of the binder samples, calculated using Eqs. (1–4), are presented in Table 2. The carbonyl index is notably higher for all bio-bitumen and MSW-bitumen samples compared to the base bitumen. This elevation is attributed to the inherently carbon-rich nature of lignin-derived biomass, which contains significant amounts of esters, ketones, aldehydes, and carboxylic acids. In the case of MSW-bitumen, the elevated carbonyl index may result from thermally degraded plastics, cellulose, and food waste components, which generate oxygenated functional groups during pyrolysis. These compounds increase the carbonyl contribution within the modified binder matrix. The sulfoxide and hydroxyl indices show comparable values across the base binder and all modified binders, suggesting minimal oxidative degradation during blending and indicating a relatively stable chemical environment. A similar hydroxyl index across samples also suggests that compatibility with the base binder is retained, minimizing the need for additional additives.

**Table 2:** FTIR Indices of the binders

	**Carbonyl**	**Sulfoxide**	**Hydroxyl**	**Aromatic**
Base binder	0.0014	0.0062	0.5771	0.0041
PN bio-bitumen	0.0066	0.0058	0.5527	0.0034
SD bio-bitumen	0.0030	0.0065	0.5188	0.0015
RS bio-bitumen	0.0045	0.0065	0.6059	0.0020
MSW-bitumen	0.0036	0.0057	0.5521	0.0254

Regarding molecular structure, the bio-bitumen samples are characterized by fatty acids, aliphatic chains, and oxygenated functional groups, but contain fewer condensed aromatic ring systems. Consequently, the aromaticity index of bio-bitumen is lower than that of the base bitumen, which may lead to lower thermal stability and reduction in resistance to oxidative aging. Interestingly, the MSW-bitumen exhibits the highest aromaticity index among all samples. This is attributed to the presence of polycyclic aromatic hydrocarbons (PAHs) formed during the pyrolysis of plastic-rich municipal solid waste, which contribute to an increased concentration of aromatic ring structures within the binder matrix. This also suggests that MSW-bitumen may display improved stiffness characteristics but may lead to compromise in low-temperature properties.

The chemical distinctions observed through FTIR analysis bear important performance implications for pavement applications. The hydrocarbon‑dominant structure of bitumen confers hydrophobicity and relative stability under thermal and oxidative loads, although the growth of sulfoxide and carbonyl groups signals susceptibility to long‑term aging (Federal Highway Administration 2024; Hofko et al. 2018). Bio‑bitumen and MSW‑bitumen have high polarity and a significant content of oxygenated functional groups, which may lead to different interfacial characteristics. These characteristics may influence the interfacial interactions between the binder and aggregates and could potentially affect moisture sensitivity and adhesion behavior at the mixture level. However, the extent of these effects would need to be confirmed through mixture-level performance testing. This is consistent with findings that pyrolysis oil additives introduce oxygen‑containing groups and reduce stiffness by increasing the mixability with asphalt (Iwański et al. 2023). The strong ester peaks imply that modified binders may be more susceptible to hydrolysis, especially under humid or alkaline conditions, and FTIR analyses of modified binders show significant decreases in ester‑related absorbance after aging (Iwański et al. 2023). Conversely, the presence of hydroxyl groups opens possibilities for reactive modification or crosslinking, aligning with evidence that modified binder aging generates additional OH‑bearing compounds such as alcohols and acids (Yang et al. 2015). Their relatively low aromatic content and high aliphatic ester composition may influence rheology, potentially lowering stiffness at intermediate temperatures unless crosslinking or polymer modification is employed (Yang et al. 2015). However, the extent to which this influences field moisture performance requires validation through mixture-level testing for moisture susceptibility assessments.

### TGA analysis

**Fig. 4:**
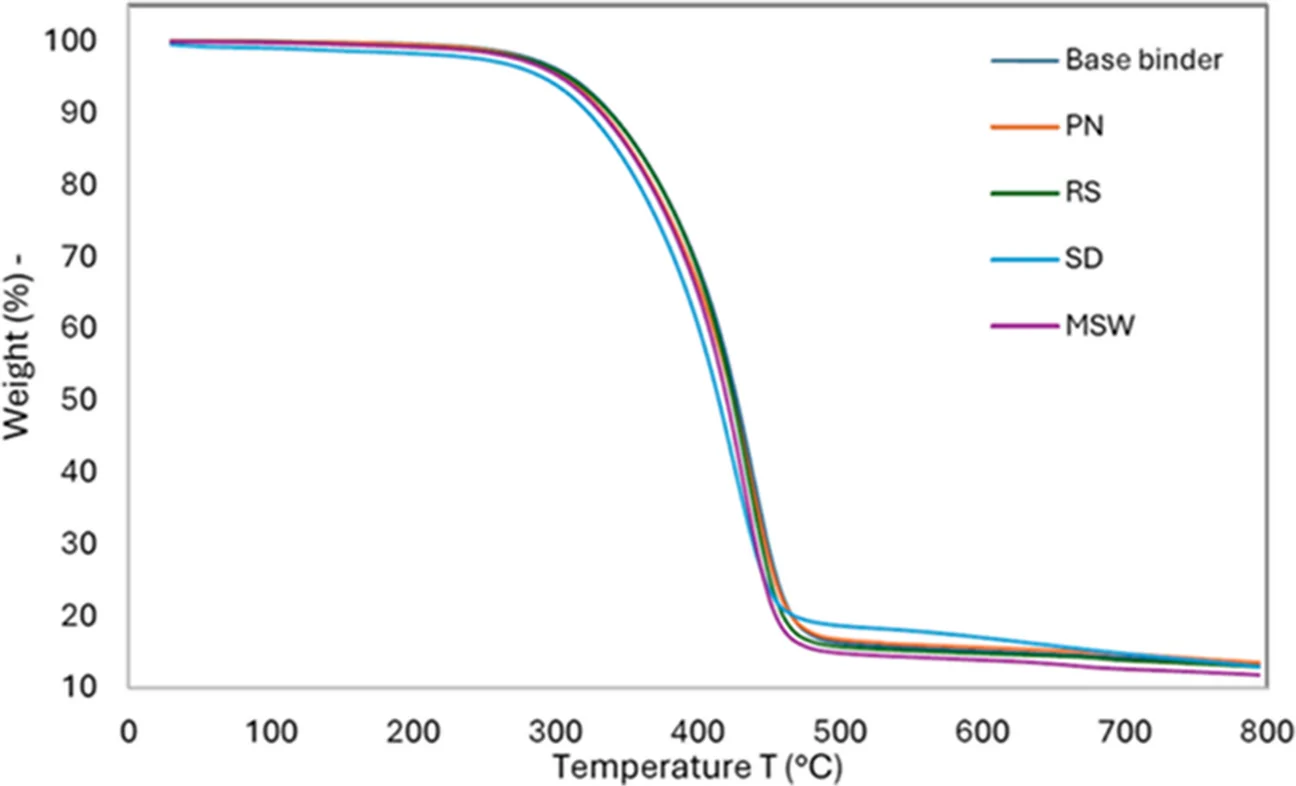
TGA decomposition graph of the bio bitumen

Figure 4 presents the TGA decomposition curves of the binder samples. The bio-bitumen binders exhibit single-step decomposition profiles, similar to that of the base bitumen. In contrast, the MSW-bitumen displays a two-step decomposition curve, with a minor mass loss event occurring around 200 °C. The overall mass loss and residue for the base bitumen were 87% and 12.9%, respectively. All modified binders exhibited mass loss values within ± 1% of the base binder, indicating comparable thermal stability in terms of total decomposition. The onset temperature for the degradation of oxygenated compounds, such as ketones, acids, and aldehydes, was lowest for the PN bio-bitumen and highest for the base bitumen. The final decomposition temperature, associated with the breakdown of aromatic and phenolic structures, was lower for the base binder and comparatively higher for all bio-bitumen samples. This indicates improved thermal stability in the aromatic fraction (asphaltenes) for the modified binders, particularly the bio-bitumen and MSW-bitumen variants (see Table 3).

**Table 3:** Results of the TGA and DTG graphs

	Base binder	PN bio bitumen	SD bio bitumen	RS bio bitumen	MSW bio bitumen
TGA graph analysis					
Mass loss (%)	87.036	86.531	87.112	87.152	88.249
Residue at 800/°C (%)	12.963	13.469	12.888	12.848	11.750
The onset temperature of decomposition $(^\circ \mathrm{C})$	239	229	226	235	228
50% mass loss T $(^\circ \mathrm{C})$ ${\mathrm{T}\mathrm{e}\mathrm{m}\mathrm{p}}_{0.5}$	426	423	414	424	419
Final temperature of decomposition $(^\circ \mathbf{C})$	484	500	484	490	493
DTG graph analysis					
${\mathrm{M}\mathrm{a}\mathrm{s}\mathrm{s} \mathrm{l}\mathrm{o}\mathrm{s}\mathrm{s} \mathrm{a}\mathrm{t} \mathrm{T}\mathrm{e}\mathrm{m}\mathrm{p}}_{\mathrm{m}\mathrm{a}\mathrm{x}}$	39.069	39.765	41.135	40.503	37.043
$$\mathrm{M}\mathrm{a}\mathrm{x}\mathrm{i}\mathrm{m}\mathrm{u}\mathrm{m} \mathrm{d}\mathrm{e}\mathrm{c}\mathrm{o}\mathrm{m}\mathrm{p}. \mathrm{T} (^\circ \mathrm{C})$$$${\mathrm{T}\mathrm{e}\mathrm{m}\mathrm{p}}_{\mathrm{m}\mathrm{a}\mathrm{x}}$$	439.23	438.49	425.349	434.297	433.565

**Fig. 5:**
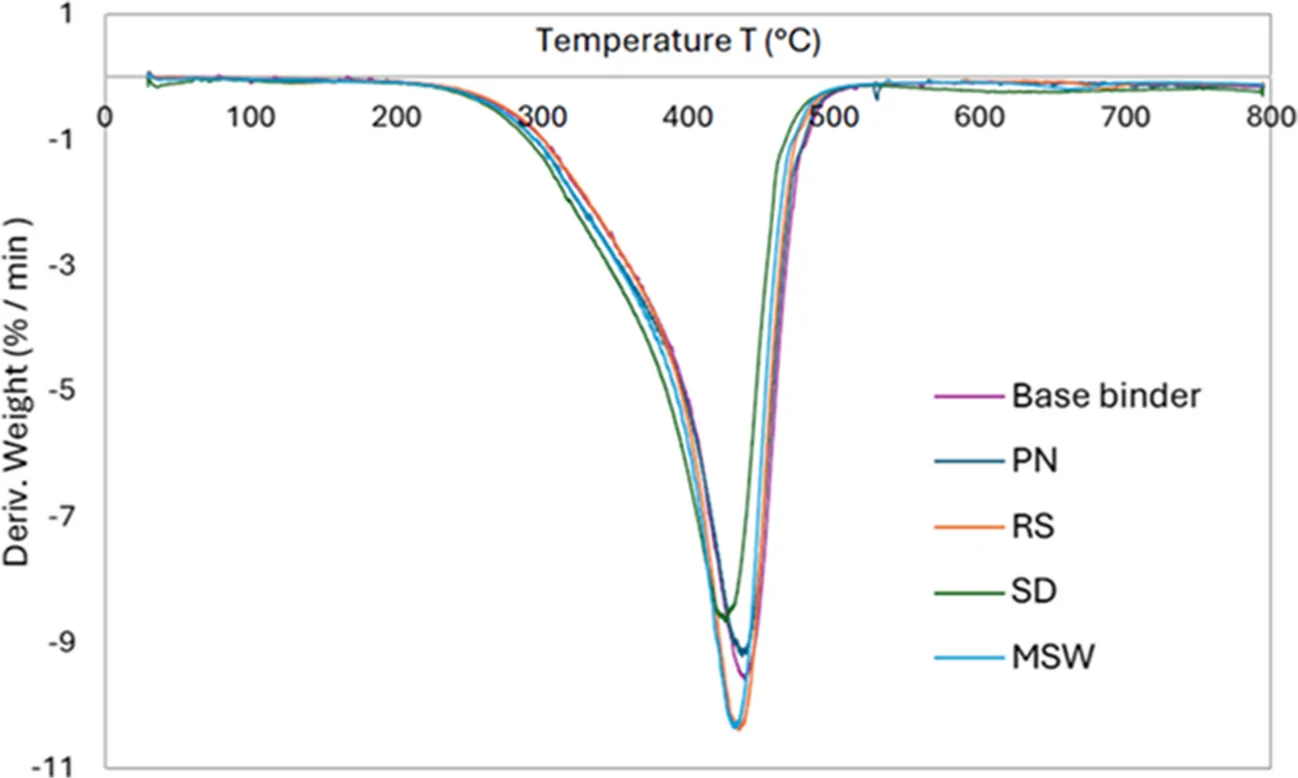
DTG graph of the TGA decomposition graph

Figure 5 shows the derivative thermogravimetric (DTG) curves derived from the TGA data. All binders exhibit a major decomposition peak corresponding to the maximum rate of thermal degradation, except for the MSW-bitumen, which displays an additional minor peak between 200 and 300 °C. This peak corresponds to the volatilization of light organic compounds, confirming a two-phase degradation process. In contrast, the single peak observed in all bio-bitumen DTG curves indicates a predominantly single-component thermal system. The presence of a single DTG peak in bio-bitumen suggests a more homogeneous thermal degradation profile, whereas the double-peak in MSW-bitumen implies the existence of multiple thermal components, likely due to the complex mixture of plastic-derived and organic fractions in MSW oil.

The maximum decomposition temperature was highest for the base bitumen and slightly lower for all bio-bitumen and MSW-bitumen samples. Among the bio-bitumens, the SD variant exhibited the highest mass loss at the peak decomposition temperature, followed by RS, with MSW-bitumen showing the lowest. A noticeable peak shift in the SD bio-bitumen indicates a reduction in thermal resilience, possibly due to a higher proportion of less stable compounds. Overall, analysis of the TGA and DTG data suggests that the PN and RS bio-bitumen samples are thermally stable, with single-phase degradation and slight peak shifts attributable to lignin-derived phenolic structures, which are known for high thermal stability. The SD bio-bitumen demonstrates lower thermal resilience due to greater peak shift and higher mass loss. The MSW-bitumen shows thermal stability comparable to the base binder but exhibits a two-component degradation pattern, reflecting the presence of both volatile and stable constituents in its structure.

### GPC analysis

The molecular weight fractions of the binder samples were determined by numerically integrating the peak areas within specified elution volume intervals of the GPC chromatograms, using Eqs. (8)–(10). The integration ranges were defined as follows: 20–24 mL for large molecular weight (LMW) fractions, 24–27 mL for medium molecular weight (MMW) fractions, and 27–30 mL for small molecular weight (SMW) fractions. The calculated molecular weight distribution parameters and corresponding molecular weight fractions for each binder are presented in Table 4, based on the GPC analysis.

**Table 4:** The parameters obtained from GPC analysis

GPC plot parameters	Base binder	PN bio bitumen		SD bio bitumen		RS bio bitumen	MSW bio bitumen
${M}_{n} (\mathrm{g}/\mathrm{m}\mathrm{o}\mathrm{l})$	1.494 × 10^3^	1.381 × 10^3^		1.500 × 10^3^		1.372 × 10^3^	1.527 × 10^3^
${M}_{w} (\mathrm{g}/\mathrm{m}\mathrm{o}\mathrm{l})$	2.459 × 10^3^	2.143 × 10^3^		2.421 × 10^3^		2.386 × 10^3^	2.576 × 10^3^
${M}_{w}/{M}_{n}$	1.646	1.552		1.614		1.739	1.687
Area	2.115 × 10^2^	1.832 × 10^2^		2.389 × 10^2^		1.769 × 10^2^	2.588 × 10^2^
η	3.000	4.204		3.746		2.166	4.800
$\mathrm{L}\mathrm{M}\mathrm{W}\mathrm{\%}$	24.7		24.2	25.0	17.9		24.7
$\mathrm{M}\mathrm{M}\mathrm{W} \mathrm{\%}$	47.6		49.6	48.7	42.7		48.2
$\mathrm{S}\mathrm{M}\mathrm{W} \mathrm{\%}$	27.7		26.2	26.3	39.4		27.1

**Fig. 6:**
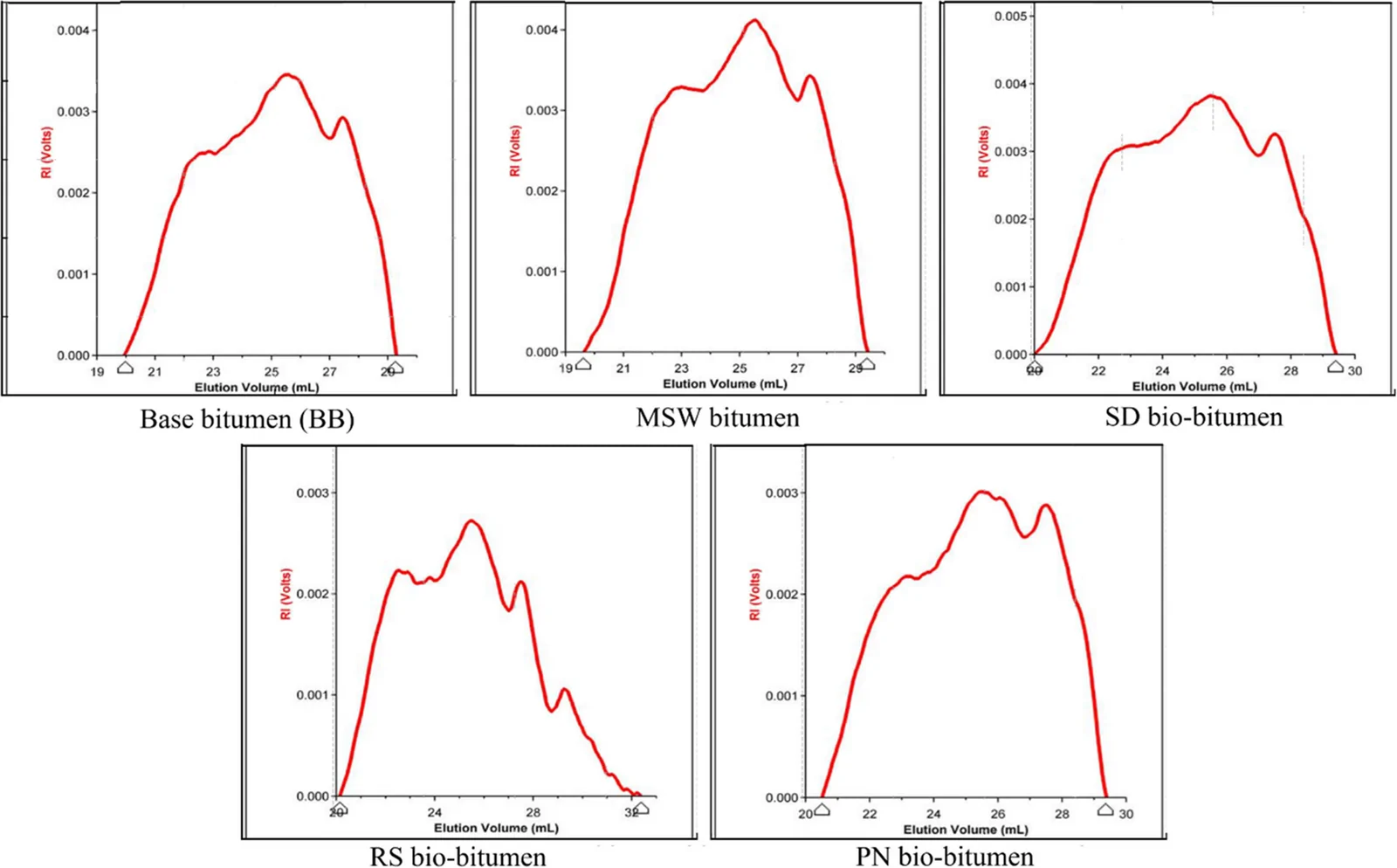
Detector response versus elution volume of the base binder and bio-bitumen

A molecular weight distribution curve, plotting retention index (RI) against elution volume (mL), was generated for all binder samples (see Fig. 6). The number-average (Mn) and weight-average (Mw) molecular weights of the base binder were found to be higher than those of all bio-bitumen samples, indicating a greater presence of high-molecular-weight polymeric and resinous species. Among the modified binders, the MSW-bitumen exhibited the highest Mn and Mw values, suggesting a molecular structure most similar to the base binder. The polydispersity indices (PDI), calculated as Mw/Mn, ranged from 1.55 to 1.74 across all samples, indicating moderately broad molecular weight distributions. The relatively low PDI values suggest uniformity in binder composition and absence of significant phase separation. Notably, the RS bio-bitumen exhibited the highest PDI, pointing to a more complex and heterogeneous molecular structure.

The area under the GPC curves for PN and RS bio-bitumen was lower than that of the base binder, implying reduced light-soluble molecular content and a higher overall polymeric concentration. This contrasts with the MSW-bitumen, which, like the base binder, exhibited a higher abundance of light molecules. Molecular fraction analysis revealed a higher proportion of LMW components in the base bitumen and MSW-bitumen, reflecting a larger asphaltene and resin fraction. The SD and PN bio-bitumen exhibited intermediate LMW fractions, suggesting a contribution from lignin-rich compounds. The RS bio-bitumen had the lowest LMW content, along with the lowest MMW and highest SMW fractions, indicative of high concentrations of light volatiles such as ketones, phenols, and esters.

While most binders, including the base binder, exhibited a trimodal molecular weight distribution, the RS bio-bitumen uniquely displayed a quad-modal distribution, further confirming its complex molecular composition. Finally, the intrinsic viscosity (*η*) values for PN bio-bitumen and MSW-bitumen were comparable to that of the base binder, indicating similar extended molecular conformations and better solvating behavior, thereby suggesting compatibility and structural alignment with conventional asphalt (see Table 4).

### Storage stability analysis

Table 5 presents the difference in softening point (∆SP) between the upper and lower sections of the test tubes, calculated using Eq. 11. All values presented in Table 5 are the mean of three replicate measurements (*n* = 3). The corresponding standard deviations were calculated through uncertainty propagation of the measurements obtained from the upper and lower sections, ensuring the reliability and statistical robustness of the reported segregation indicators. All bio-bitumen samples exhibit good storage stability, with ∆SP values less than |2.5 °C|, which is within the acceptable limit. Notably, the ∆SP values of the bio-bitumen are slightly higher than those of the base binder, with the PN and MSW variants showing the least increase in ∆SP, suggesting better homogeneity and phase compatibility upon thermal conditioning. To further assess segregation, the complex shear modulus (G*) of the upper and lower sections was measured, and the segregation ratio (SR) was calculated using Eq. 12. For all bio-bitumen samples, the SR was found to be ≤|1.2|, again falling within the acceptable threshold for storage-stable binders.

**Table 5:** Segregation index from the softening point test and complex shear modulus

	Base binder	PN bio bitumen	SD bio bitumen	RS bio bitumen	MSW bio bitumen
∆SP (°C)	0.05 ± 0.04	0.2 ± 0.09	0.3 ± 0.14	0.6 ± 0.15	0.2 ± 0.12
SR	0.94 ± 0.01	1.07 ± 0.03	1.13 ± 0.02	1.07 ± 0.02	1.10 ± 0.02

Interestingly, Table 5 shows that the base binder has an SR value < 1, whereas all bio-bitumen samples have SR values > 1. This discrepancy is likely due to the presence of lighter fractions in the bio-bitumen, which may tend to migrate toward the upper section during the storage stability test. These light components may also undergo oxidation during thermal conditioning, further contributing to the observed SR increase. Despite this, all segregation indicators, both ∆SP and SR, remain within acceptable limits, confirming that all bio-bitumen samples are storage-stable and suitable for practical applications. The storage stability results, in conjunction with the GPC intrinsic viscosity data and single-phase TGA decomposition profiles, collectively confirm that the compatibility of pyrolysis oils with the base bitumen extends beyond chemical similarity observed through FTIR, encompassing physical phase stability and molecular-level homogeneity. These converging lines of evidence from multiple analytical techniques provide a robust basis for the compatibility assessment reported in this study.

### Rheological analysis

**Fig. 7:**
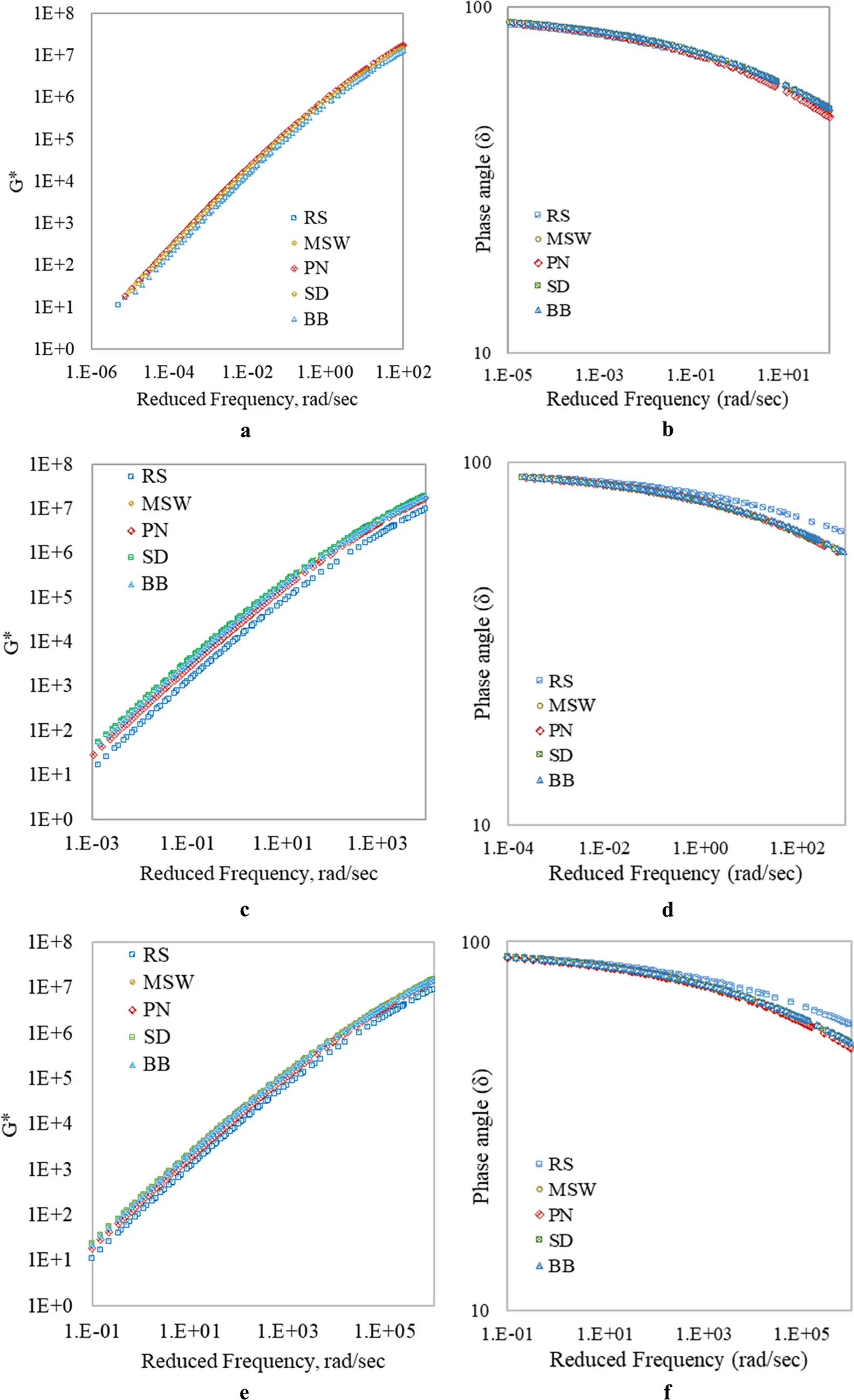
Master curves of the binders

Figure 7a, c, and d presents the master curves of the complex shear modulus (G*) at three different reference temperatures 20 °C, 40 °C, and 70 °C of the base bitumen and the modified bitumen, respectively. The reference temperatures were strategically selected to characterize the stiffness response of the binders across the representative range of operational pavement temperature regimes. All the binders exhibit the expected viscoelastic trend of an increase in the G* with the increase in the reduced frequency and a reduction in phase angle. The G* and *δ* values of the binders are compared at 10 rad/s. It is found that similar trends of the G* are observed for all the modified binders, irrespective of the temperatures under study. For further analysis, the phase angle (*δ*) master curves are constructed using Eqs. (13) and (15). Figure 7b, d, and f presents the master curves of the binders at three different reference temperatures: 20 °C, 40 °C, and 70 °C of the base bitumen and the modified bitumen, respectively. All binders display a typical shift from viscous behavior at low frequencies to elastic behavior at high frequencies. Compared to the base binder, all modified binders exhibit increased *δ* values, indicating enhanced shear deformation and reduction in elastic response. The G* and *δ* values of the binder measured at 10 rad/s is presented in Table 6.

**Table 6:** Comparison of complex shear modulus and phase angle at 10 rad/s

Binders	Complex shear modulus			Phase angle		
	20°	40°	70°	20°	40°	70°
BB	8.5 × 10^5^	2.8 × 10^5^	5.5 × 10^5^	45°	55°	72°
PN	7.5 × 10^5^	2.5 × 10^5^	4.8 × 10^5^	47°	57°	74°
RS	6.0 × 10^5^	2.0 × 10^5^	3.5 × 10^5^	50°	59°	76°
SD	6.8 × 10^5^	2.2 × 10^5^	4.0 × 10^5^	48°	58°	75°
MSW	5.5 × 10^5^	1.7 × 10^5^	2.8 × 10^5^	52°	61°	78°

At 20 °C, all modified binders show a reduction in stiffness compared to the base binder, PN (−12%), RS (−29%), SD (−20%), and MSW (−35%). Correspondingly, their phase angles increase by + 2° to + 7°, indicating a more viscous character. At 40 °C, the trend persists with PN (−11%), RS (−29%), SD (−21%), and MSW (−39%) having lower G* than base bitumen, while *δ* rises by + 2° to + 6°. At 70 °C, the decrease in G* is more pronounced, with PN (−13%), RS (−36%), SD (−27%), and MSW (−49%) compared to base bitumen, while *δ* increases further by + 2° to + 6°. Overall, the bio-binders consistently reduce stiffness and increase phase angle relative to the base binder, with MSW showing the greatest softening and viscous shift, while PN remains the closest in performance to base bitumen. The decrease in G* and increase in δ of the bio-binders compared to the base binder indicate a reduction in stiffness and a shift toward more viscous behavior. This means the modified binders may exhibit better workability and flexibility but at the cost of reduced resistance to permanent deformation, particularly at higher service temperatures. Among them, PN shows properties closest to the base binder, while MSW shows the greatest softening, suggesting it may be less suitable for high-temperature pavement performance. In summary, the PN bio-bitumen outperforms among the bio-bitumens in viscoelastic behavior, combining high stiffness with low shear deformation, making it the most promising modifier. Conversely, the MSW bio-bitumen consistently exhibits higher δ values and lower G*, identifying it as the softest binder with the greatest vulnerability to temperature and shear-induced deformation.

### Aging analysis

**Fig. 8:**
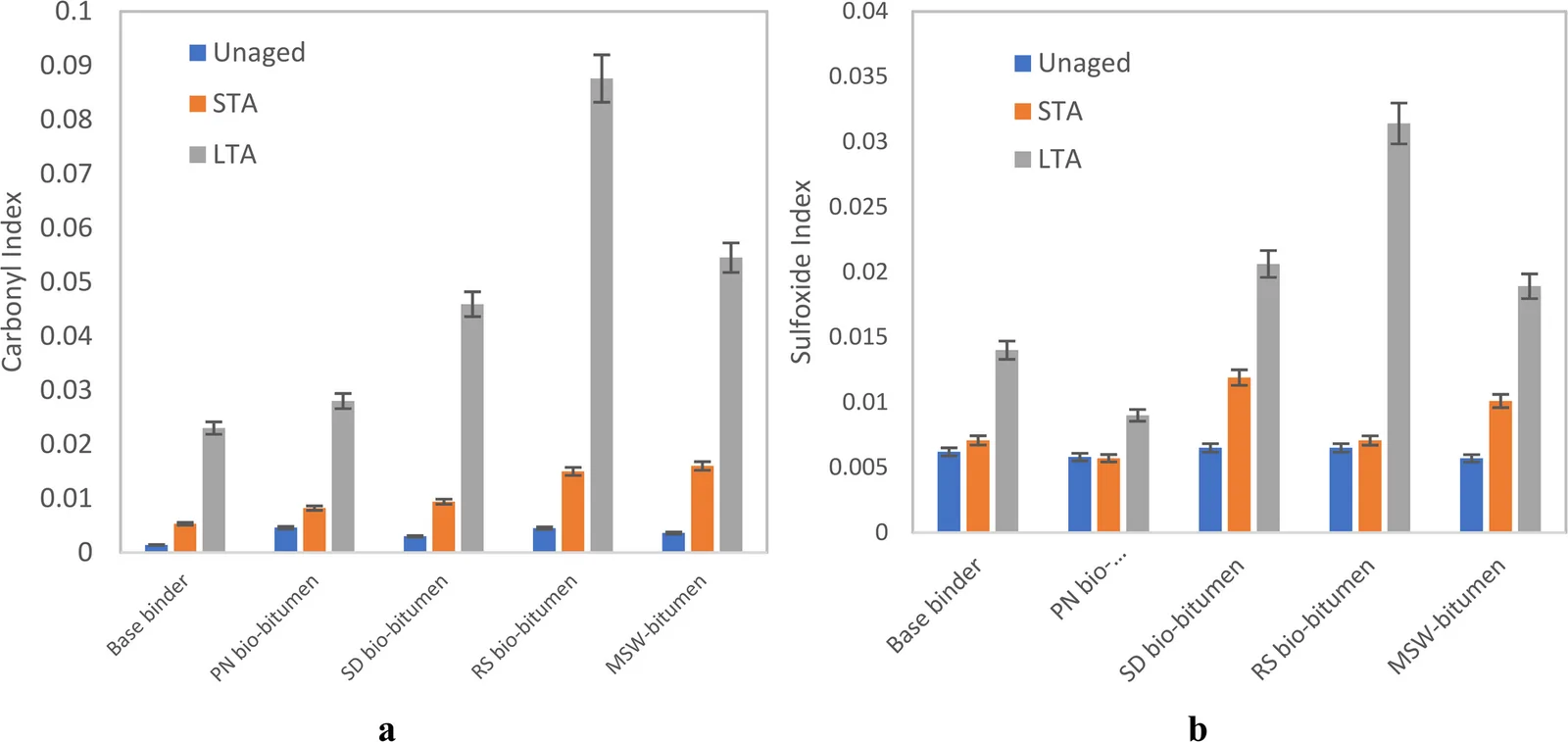
Aging indices

The FTIR aging indices presented in Fig. 8 were determined from three independent replicate measurements (*n* = 3), and the reported values represent the mean response. The relatively small standard deviations observed for most measurements indicate good repeatability of the FTIR test results and provide confidence in the trends identified among the binder types. The carbonyl index shows a consistent increase in the aging index for all binders, confirming carbonyl formation as a primary oxidation pathway during bitumen aging (Fig. 8a). The base binder exhibits the lowest carbonyl growth across all stages and therefore serves as the oxidation‑stable reference. Among the modified binders, PN bio‑bitumen displays a modest increase under STA and a slightly higher LTA value than the base binder, indicating a moderate increase in long‑term oxidation but within acceptable limits. SD and MSW bio‑bitumen show substantially higher carbonyl accumulation during LTA, approximately 2 to 2.5 times the base binder, indicating reduced resistance to long‑term thermo‑oxidative aging. RS bio‑bitumen exhibits the highest carbonyl index under all aging states, with its LTA carbonyl value far exceeding those of the base and other modified binders. This clearly identifies RS as the most oxidation‑prone binder, with significant susceptibility to structural changes associated with carbonyl group formation.

The sulfoxide index follows a similar trend, increasing progressively with aging severity (Fig. 8b). The base binder again shows the lowest sulfoxide development under LTA. Notably, PN bio‑bitumen exhibits a lower LTA sulfoxide index than the base binder, suggesting a reduced tendency toward sulfoxidation despite its moderate carbonyl increase. This indicates a favorable oxidative aging profile, with PN showing suppressed polarity increase, which is beneficial for long‑term flexibility. MSW and SD bio‑bitumen show significantly elevated sulfoxide growth under LTA, correlating with increased formation of polar oxygenated species and higher susceptibility to oxidative hardening. RS bio‑bitumen again demonstrates the highest sulfoxide index among all binders, confirming its strong tendency toward oxidation via both carbonyl and sulfoxide pathways.

PN bio‑bitumen shows the most favorable aging behavior among the modified binders. Its low sulfoxide formation and moderate carbonyl increase imply slower hardening, lower polarity build‑up, and better retention of viscoelastic balance during service life. MSW bio‑bitumen demonstrates moderate oxidation susceptibility. While not as stable as PN, its oxidative growth patterns suggest manageable aging‑related stiffening. SD bio‑bitumen exhibits significantly higher long‑term carbonyl and sulfoxide indices, indicating accelerated oxidative hardening, increased viscosity growth, and a greater risk of fatigue and thermal cracking as aging progresses. RS bio-bitumen, showing the highest values for both carbonyl and sulfoxide indices, exhibits the greatest oxidative hardening tendency at the binder level. The elevated carbonyl and sulfoxide indices after RTFO and PAV aging reflect increased oxidation and polarity buildup, which typically results in greater binder stiffness, reduced molecular mobility, and higher susceptibility to embrittlement. These chemical changes are consistent with the rheological observations, where binders exhibiting lower growth in FTIR aging indices, particularly PN bio-bitumen, also demonstrated better retention of viscoelastic properties after aging. In contrast, RS and SD bio-bitumen showed higher oxidation indices, consistent with their greater susceptibility to aging-related rheological deterioration. This underlines that oxidative aging behavior varies significantly across binder types, and PN bio‑bitumen stands out as the most promising modification for enhancing long‑term performance. Confirmation of field-scale embrittlement and associated cracking performance would nonetheless require mixture-level fatigue and low-temperature cracking tests.

## Discussions

The compositional analysis of the feedstocks revealed clear differences in their suitability for bitumen modification. PN feedstock exhibited the most favorable profile, combining the lowest *O*/*C* ratio, a favorable *H*/*C* ratio, low ash content, and the highest HHV. Collectively, these characteristics indicate lower polarity and reduced inorganic content, which are beneficial for binder compatibility and aging resistance (Anshu et al. 2024; Zhou et al. 2021). MSW emerged as a viable secondary option; however, its elevated ash content and sulfur presence necessitate pretreatment, such as deashing, before binder application. In contrast, RS and SD exhibited substantially higher oxygen contents and ash levels, indicating greater oxygenation and inorganic content. As a result, extensive upgrading, including deashing, esterification, or mild hydrodeoxygenation, would be required to achieve adequate compatibility, stability, and moisture resistance with petroleum binders (Tabaković et al. 2023; Lu and Gu 2022).

FTIR analysis of the pyrolysis oils confirmed these compositional trends at the molecular level. PN oil exhibited a balanced chemical profile with moderate O–H and C = O intensities, distinct aromatic bands, and strong aliphatic C–H stretching vibrations, indicating lower polarity and enhanced compatibility with hydrophobic bitumen (Yao et al. 2015; Dong et al. 2018). Combined with its high carbon and hydrogen contents and low ash level, these characteristics make PN oil the most promising candidate for asphalt binder modification. By comparison, RS oil displayed dominant O–H, C = O, and C–O absorption bands, confirming its high oxygenation and polarity, whereas MSW oil exhibited stronger aliphatic C–H bands, reflecting its more hydrocarbon-like composition derived from plastic-rich waste streams.

At the binder level, both bio-bitumen and MSW-bitumen exhibited considerably higher polarity than the base bitumen, as evidenced by strong ester, alcohol, and C–O/O–H absorptions, including a pronounced carbonyl peak at 1740 cm⁻^1^. In contrast, the base bitumen remained predominantly hydrocarbon-based, with oxygenated functional groups arising mainly from oxidative aging. Consequently, the carbonyl absorption observed in the modified binders is an intrinsic feature of their composition rather than a marker of oxidation, whereas in the base bitumen, it is primarily associated with aging-induced oxidation (Iwański et al. 2023; Yang et al. 2015). Among all modified binders, PN bio-bitumen exhibited peak intensities most comparable to those of the base bitumen, suggesting superior compatibility and lower polarity than the more hydroxyl-rich and moisture-sensitive RS and SD variants. The absence of major new chemical bonds in all modified binders further confirms that bio-oil incorporation is predominantly a physical modification process rather than a chemical one (Guo et al. 2024; Wu and Hou 2024).

The elevated carbonyl indices observed in bio-bitumen and MSW-bitumen indicate enrichment in oxygenated functional groups, including fatty acids and aliphatic chains originating from biomass- and waste-derived pyrolysis products. The lower aromaticity of bio-bitumen, resulting from a reduced concentration of condensed aromatic ring systems, is associated with lower thermal stability and decreased resistance to oxidative aging (Al-Sabaeei et al. 2020). Conversely, the higher aromaticity of MSW-bitumen, attributed to PAHs generated during the pyrolysis of plastic-rich waste, may contribute to increased stiffness but could adversely affect low-temperature flexibility (Hall and White 2021). These findings are supported by GPC analysis, which identified lignin-rich fractions together with light aromatic and heavy oil compounds within the modified binders (Li et al. 2022).

Thermal analysis showed that all bio-bitumen samples, except MSW-bitumen, exhibited single-step decomposition profiles characteristic of homogeneous single-component thermal systems. PN and RS bio-bitumen displayed thermal stability comparable to that of the base binder, with only minor shifts in decomposition peaks attributable to lignin-derived phenolic structures known for their high thermal stability (Lu and Gu 2022; Mehta and Saboo 2025). In contrast, SD bio-bitumen exhibited lower thermal resilience, as indicated by a greater peak shift and increased mass loss at the maximum decomposition temperature. The two-step decomposition behavior observed in MSW-bitumen reflects the coexistence of volatile and thermally stable constituents derived from the complex mixture of plastic-derived and organic fractions present in MSW oil, consistent with the thermal behavior reported for polymeric waste-modified binders in the literature (Ephraim et al. 2023).

GPC-based molecular weight distribution analysis revealed that MSW-bitumen and the base bitumen contained similar proportions of high-molecular-weight fractions, indicating comparable levels of asphaltenes and resins. Among the modified binders, PN bio-bitumen and MSW-bitumen exhibited intrinsic viscosity values and molecular conformations most similar to those of the base binder, suggesting favorable solvency and structural compatibility (Negulescu and Balamurugan 2013; Li et al. 2022). In contrast, RS bio-bitumen displayed a distinctive quad-modal molecular weight distribution, indicating a more complex and heterogeneous molecular structure that may contribute to its inferior rheological behavior and aging resistance relative to the other modified binders.

Considering both ΔSP and SR results, all bio-bitumen modified binders demonstrated acceptable storage stability and phase compatibility. PN and MSW bio-bitumens exhibited the lowest ΔSP values (0.2 °C), reflecting the highest degree of homogeneity. In terms of SR, PN and RS bio-bitumens showed values closest to unity (1.07), suggesting slightly better phase compatibility than the MSW (1.10) and SD (1.13) binders. Although RS bio-bitumen presented the highest ΔSP value (0.6 °C), both its ΔSP and SR remained within acceptable limits. Overall, the results confirm that all modified binders maintained satisfactory storage stability and compatibility, with PN bio-bitumen showing the most balanced performance based on both indicators (Fini et al. 2017; Zhang et al. 2018).

Rheological analysis revealed that all modified binders exhibited lower G* values and higher *δ* than the base binder, indicating a shift toward more viscous behavior (Fini et al. 2012; Dong et al. 2018). Among the modified binders, PN bio-bitumen demonstrated the most balanced viscoelastic performance, maintaining higher stiffness and lower phase angles that were closest to those of the base binder. In contrast, MSW-bitumen consistently exhibited the lowest stiffness and highest phase angles, particularly at elevated temperatures, identifying it as the most deformation-prone binder evaluated in this study. These rheological trends are consistent with the compositional and molecular characteristics identified through FTIR and GPC analyses, which showed that PN oil possesses the closest chemical affinity to conventional petroleum binders.

Aging analysis showed that all modified binders experienced progressive increases in carbonyl and sulfoxide indices after RTFO and PAV conditioning, confirming oxidative hardening as the primary aging mechanism (He et al. 2023; Hofko et al. 2018). The growth of these indices indicates increased oxidation, leading to higher stiffness and greater susceptibility to embrittlement. PN bio-bitumen exhibited the most favorable aging behavior, with moderate carbonyl growth and the lowest sulfoxide formation, suggesting slower oxidative hardening and better retention of viscoelastic properties. This finding agrees with Park et al (2022), who reported that phenol-rich bio-oils can retard bitumen oxidation. In contrast, RS bio-bitumen showed the highest carbonyl and sulfoxide indices, indicating the greatest susceptibility to oxidative degradation, while SD and MSW bio-bitumens exhibited intermediate aging behavior.

## Recommendations

Based on the findings of this study, the following recommendations are proposed:

- PN bio-oil is identified as the most technically promising candidate for asphalt binder modification and is recommended for further investigation at the mixture level. Future studies should evaluate PN bio-bitumen performance across varying replacement percentages, ranging from 5% to higher substitution levels, to establish the optimum substitution rate that balances rheological performance with workability and aging resistance.
- RS and SD bio-oils, characterized by high oxygen content and elevated ash levels, require significant upgrading before practical application. Pretreatment strategies such as deashing, esterification, and mild hydrodeoxygenation are recommended to reduce oxygenated functional group content, lower polarity, and improve compatibility with petroleum binders. Without such upgrading, the accelerated oxidative hardening and inferior rheological performance observed for these bio-oils limit their direct applicability as asphalt modifiers.
- MSW-derived pyrolysis oil demonstrated acceptable binder modification characteristics at the laboratory scale. However, comprehensive contaminant characterization is strongly recommended before any field-scale application. Analyses including ICP-MS or XRF for heavy metals and halogen analysis for chlorine-containing species should be conducted to assess the environmental suitability and regulatory compliance of MSW-derived oils in pavement applications. Leaching assessments under simulated field conditions are also recommended to evaluate potential environmental risks associated with long-term pavement service.
- Mixture-level mechanical testing is recommended as the next step to validate the binder-level findings and assess the practical pavement applicability of the investigated bio-binders.
- Future studies should include life cycle assessment (LCA), energy consumption, and carbon footprint analyses to quantify the environmental benefits of these bio-binders relative to conventional petroleum-based binders and support their large-scale adoption.

## Conclusion

This study evaluated asphalt binders modified with pyrolysis oils derived from pine needle (PN), rice straw (RS), sawdust (SD), and municipal solid waste (MSW) through comprehensive chemical, molecular, thermal, rheological, storage stability, and aging analyses. The results demonstrate that the performance of pyrolysis oil modified binders is strongly dependent on feedstock characteristics. Among the investigated sources, PN bio-bitumen exhibited the closest similarity to the base binder, showing favorable molecular composition, thermal stability, rheological behavior, storage stability, and resistance to oxidative aging. MSW-bitumen showed acceptable performance, although additional contaminant characterization is recommended before practical implementation. In contrast, RS and SD bio-oils exhibited higher oxygenation and greater aging susceptibility. Overall, PN-derived pyrolysis oil emerged as the most promising modifier, highlighting the importance of feedstock selection in the development of sustainable bio-bitumen technologies.

## Data Availability

All data generated or analyzed during this study are included in this published article.
